# Footprints of ancient-balanced polymorphisms in genetic variation data from closely related species

**DOI:** 10.1111/evo.12567

**Published:** 2015-01-16

**Authors:** Ziyue Gao, Molly Przeworski, Guy Sella

**Affiliations:** 1 Committee on Genetics, Genomics and Systems Biology, University of ChicagoChicago, Illinois, 60637; 3 Department of Biological Sciences, Columbia UniversityNew York, New York, 10027; 4 Department of Systems Biology, Columbia UniversityNew York, New York, 10032

**Keywords:** Ancient genetic variation, balancing selection, genome scan for selection, trans-species polymorphism

## Abstract

When long-lasting, balancing selection can lead to “trans-species” polymorphisms that are shared by two or more species identical by descent. In such cases, the gene genealogy at the selected site clusters by allele instead of by species, and nearby neutral sites also have unusual genealogies because of linkage. While this scenario is expected to leave discernible footprints in genetic variation data, the specific patterns remain poorly characterized. Motivated by recent findings in primates, we focus on the case of a biallelic polymorphism under ancient balancing selection and derive approximations for summaries of the polymorphism data from two species. Specifically, we characterize the length of the segment that carries most of the footprints, the expected number of shared neutral single nucleotide polymorphisms (SNPs), and the patterns of allelic associations among them. We confirm the accuracy of our approximations by coalescent simulations. We further show that for humans and chimpanzees—more generally, for pairs of species with low genetic diversity levels—these patterns are highly unlikely to be generated by neutral recurrent mutations. We discuss the implications for the design and interpretation of genome scans for ancient balanced polymorphisms in primates and other taxa.

Balancing selection is a mode of adaptation that leads to the presence of more than one allele in the population at a given time. Although balancing selection is often assumed to be the result of heterozygote advantage (also known as overdominance), possible sources are diverse and include negative frequency-dependent selection and temporally or spatially heterogeneous selection (Fisher 1922; Levene 1953; Nagylaki 1975; Wilson and Turelli 1986). The common outcome of these diverse modes is the persistence of genetic variation beyond what is expected from genetic drift (or directional selection) alone (Dobzhansky 1970; Kimura 1983).

Balancing selection leaves discernible footprints in genetic variation data (Charlesworth 2006). In particular, the effects of an old balanced polymorphism on variation data within a species are well understood (e.g., Kaplan et al. 1988). A site under long-lasting balancing selection has a deeper genealogy than expected under neutrality, with long internal branches. Because of linkage, the genealogies at nearby sites will have similar properties. As a result, an old balanced polymorphism leads to higher diversity and more intermediate frequency alleles at linked neutral sites. These considerations suggest that targets of old balancing selection could be identified from their footprints in genetic variation data (Charlesworth 2006). A challenge, however, is that these footprints can also be produced by neutral processes alone, raising the concern of a high false discovery rate (FDR; Bubb et al. 2006; Charlesworth 2006; DeGiorgio et al. 2014).

When balancing selection is sufficiently long-lasting that it predates the split of two or more species, it may lead to a “trans-species polymorphism” that is shared by two or more species identical by descent (Figueroa et al. 1988). If two species are sufficiently diverged that no shared variation is expected by chance, the persistence of ancestral variation to the present in both species is a distinctive signature of balancing selection (Wiuf et al. 2004). For that reason, a trans-species polymorphism is considered the least equivocal evidence for ancient balancing selection (Charlesworth 2006).

Such trans-species polymorphisms are thought to be extremely rare, because the long-term persistence of a polymorphism in two or more species requires a long-lasting and relatively strong selection pressure to prevent the loss of alleles by genetic drift, as well as a low turnover rate of selected alleles. Until recently, only a handful of convincing examples have been reported: the opsin polymorphism in New World monkeys, the self-incompatibility genes (SI) in plants and fungi, the A/B polymorphism in the ABO blood group in primates, and the major histocompatibility complex (MHC) in vertebrates (Figueroa et al. 1988; Ioerger et al. 1990; Clark 1997; Surridge and Mundy 2002; Charlesworth et al. 2006; Ségurel et al. 2012). Even for these few cases, the functional variants are not always well characterized and are usually inferred to be distinct based on the clustering of the phylogenetic tree of DNA sequences.

Although all these cases were identified by candidate gene approaches, genome-wide variation data now provide the opportunity to scan for instances across the entire genome and hence to learn about ancient balancing selection more comprehensively. Application of this approach identified at least six trans-species polymorphisms that have been maintained in humans and chimpanzees to the present without allelic turnover (Leffler et al. 2013). These findings, since supported by a second study in humans (Rasmussen et al. 2014), suggest that trans-species polymorphisms are not singular anomalies, and that there are likely additional cases of variation maintained for millions of years, of which some remain unrecognized.

Interestingly, like the A/B polymorphism at *ABO*, but unlike other known cases, the balanced polymorphisms recently identified in humans appear to be biallelic. The evolutionary mechanism for their maintenance is unknown, but seems unlikely to be overdominance: stable overdominance requires strong selection and inflicts a large segregation load (Kojima 1971; Golding 1992) and in this case, the selective pressure is likely to lead to the evolution of greater plasticity or a duplication (as has happened twice for the opsin polymorphism in primates; Hunt et al. 1998). Instead, temporally fluctuating selective pressures or negative frequency-dependent selection may be more plausible mechanisms (Stahl et al. 1999; Tellier and Brown 2007), consistent with the tentative evidence linking the recently identified trans-species polymorphisms in humans to host–pathogen interactions (Leffler et al. 2013; Ségurel et al. 2013). To understand why genetic variation is actively maintained by natural selection over such long time periods, we need to identify as many cases of ancient balanced polymorphisms as possible and dissect their molecular functions.

In principle, targets of ancient balancing selection can be identified directly from genetic variation data of two or more species by scanning for shared polymorphisms (polymorphisms at homologous positions with the exactly same segregating alleles; e.g., Asthana et al. 2005). A major challenge is that many single nucleotide polymorphisms (SNPs) shared between species likely arise from recurrent mutations (i.e., independent occurrences of the same mutation in both species): indeed, between humans and chimpanzees, shared SNPs are enriched for hypermutable CpG sites and have an allele frequency spectrum similar to nonshared SNPs (Asthana et al. 2005; Leffler et al. 2013). Although the resulting shared polymorphism mimics a trans-species polymorphism, the underlying gene genealogy is markedly different from that of a trans-species polymorphism. The unusual depth and distinctive topology of the genealogies around a trans-species polymorphism therefore provide a potential approach to distinguish these two cases.

Motivated by these considerations, we consider a biallelic trans-species polymorphism maintained without turnover by balancing selection and describe the underlying genealogical structures expected at linked sites. Previous work indicated that the signal of a single trans-species polymorphism is restricted to a very short segment, because of the erosion due to recombination events in the two species (Wiuf et al. 2004). However, as we show, within this short segment, there may still be compelling evidence for ancient balancing selection. Specifically, we derive approximations for three summaries that reflect distinct aspects of this genealogical structure and are sensitive and specific to the presence of a trans-species polymorphism: the distribution of the length of the segment that carries the signals, the expected number of shared neutral SNPs, and the expected linkage disequilibrium (LD) pattern among them. We confirm the accuracy of the approximations by coalescent simulations. Although in principle our derivations can be applied to any pair of related species, we focus on humans and chimpanzees, because this pair of species is well suited for scans for ancient balancing selection (see Discussion); moreover, because multiple examples of trans-species polymorphisms have been found in this species pair (Leffler et al. 2013), we can use our results to gain further insights into their evolutionary history.

## Results

### THE MODEL

We consider a simple demographic scenario in which an ancestral species splits into two species *T* generations ago, with no subsequent gene flow between them. For simplicity, we assume panmixia and constant population sizes for the ancestral (*N_a_*) and descendant species (*N_e_*). We consider an ancestral dimorphism under balancing selection where allele A1 is maintained at constant equilibrium frequency *p* and allele A2 at *q* = 1 − *p*, and assume that the two alleles reach their equilibrium frequencies immediately after the balanced polymorphism arose. In our model, there is no subsequent mutation at the selected site, so there is no reverse mutation or allele turnover and, consequently, all chromosomes carrying the same allele (even from different species) are identical by descent. When the ancestral species splits into two, this selected polymorphism is passed down into each descendant species and maintained by balancing selection at constant frequency until the present, resulting in a polymorphism shared between the species.

**Figure 1:**
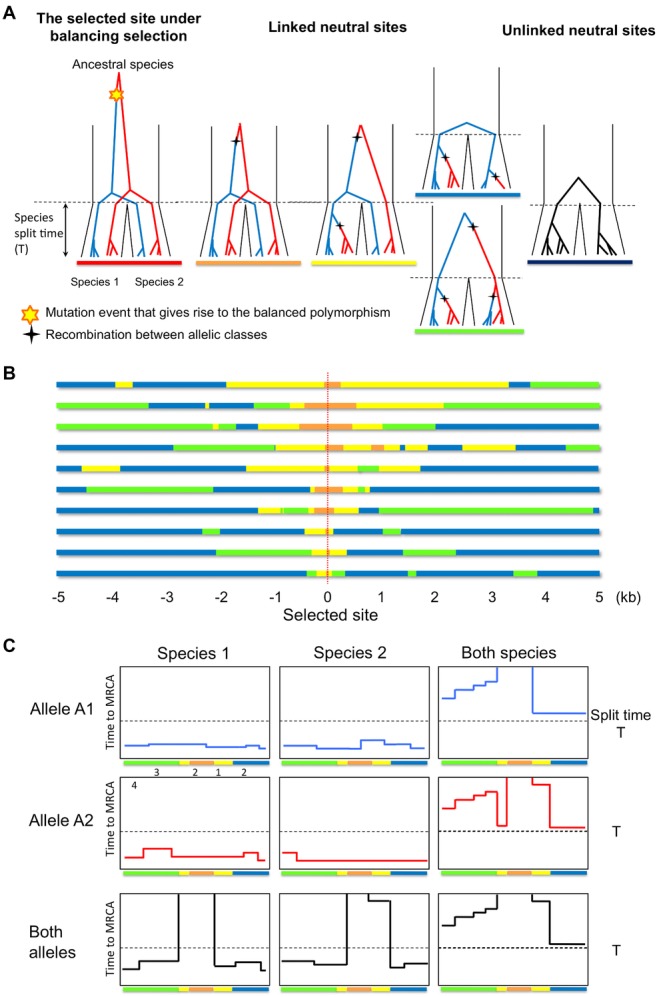
Sites linked to a balanced trans-species polymorphism have unusual genealogies. (A) The trees are ordered by the distance from the selected site. Blue and red lines represent lineages from the two allelic classes, respectively. (B) The state of each segment in ten simulation replicates. Each bar represents a 10 kb region centered on a trans-species balanced polymorphism (red dotted line). The color of the bar indicates the genealogical state of each segment (same as in A). Parameters were chosen to be plausible for humans and chimpanzees: *N* = 10,000, *N_a_* = 50,000, *T* = 160,000, *p* = 0.5, and *r* = 1.25 cM/Mb (see Supporting Information). (C) Summary of the coalescent time for a single realization of a segment carrying a balanced trans-species polymorphism. The sample consists of 20 lineages in total, five from each allelic class in each species. Each plot shows the time to the MRCA for a specific subset of the 20 lineages indicated on the top and left. The selected site is located in the center of the segment.

Throughout, we consider *T* much larger than *N_e_*. This condition is needed for trans-species polymorphisms to be a clear-cut manifestation of balancing selection, as otherwise a considerable proportion of the trans-species polymorphisms in the genome will be due to neutral incomplete lineage sorting (ILS; Wiuf et al. 2004). Indeed, a neutral trans-species polymorphism will occur if three conditions are met: (1) at least two lineages from each species do not coalesce by the time of the split (throughout the article, we measure time backwards, unless otherwise specified); (2) the first coalescent event in the ancestral species is not between lineages from the same descendant species; (3) there is a mutation in the appropriate lineage(s) to give rise to a shared polymorphism. Under our demographic assumptions, the probability of a neutral trans-species polymorphism is mainly determined by the probability of the first condition, which is approximately (*e*^−^*^T^*^/^*^2Ne^*) × (*e*^−^*^T^*^/^*^2Ne^*) (Wiuf et al. 2004). This simple derivation indicates that trans-species polymorphism is highly unlikely if the species split sufficiently long ago (in units of 2*N_e_* generations). An implication is that at a neutral site unlinked to a site under balancing selection, the local gene tree should cluster by species (Fig.1A).

### THE GENEALOGIES OF SITES AROUND A BALANCED TRANS-SPECIES POLYMORPHISM

We model the genealogy around a balanced trans-species polymorphism by using the framework of Hudson and Kaplan (Hudson and Kaplan 1988). The Hudson–Kaplan model is a form of structured coalescent (Nordborg 1997), in which allelic classes at the selected site are analogous to subpopulations: sequences carrying the same allele are exchangeable, whereas sequences carrying different alleles cannot coalesce unless they “migrate” into the same allelic group by undergoing between-class recombination. Henceforth, we assume all recombination to be *crossing over* (ignoring gene conversion without exchange of flanking markers) and use “recombination” to denote “between-class recombination,” unless otherwise specified.

The gene trees at linked neutral sites change with the genetic map distance from the selected site (Fig.1A; henceforth, “genetic distance” means “genetic map distance” that is usually measured in Morgans or centi-Morgans). At a tightly linked site, the tree has the same topology as the selected site. Notably, lineages carrying the same selected allele coalesce before lineages carrying different selected alleles and the tree clusters by allele instead of by species (the orange topology in Fig.1A). We term the segment with this topology the *ancestral segment*. At a site a little farther from the selected site, the probability of recombination between this neutral site and the selected site is larger, so a recombination event may occur in one of the two descendant species before the split. Assuming, without loss of generality, that the recombination event takes place in species 1, all lineages in species 1 will carry the same allele after the recombination and thus are closely related to each other. However, this is not true for species 2: some lineages from species 2 are more closely related to lineages from species 1 than to other lineages from species 2 (the yellow topology in Fig.1A). At a site farther away from the selected site, recombination events may occur in both species before the split, so the tree will reflect the species relationship; however, the coalescent time between the two lineages in the ancestral population varies greatly, depending on whether the allelic identities of them are the same or different (the green and blue topologies in Fig.1A).

A natural way of examining trans-specificity of a shared polymorphism is therefore to build a phylogenetic tree of sequences from both species and test if there is strong support for a tree that clusters by allele. But over what window size? The window must include the information in the ancestral segment; but it cannot be too large or it will incorporate segments with other genealogical topologies that will dilute the signal of trans-specificity (Nordborg and Innan 2003). It is also important to understand which features of the data indicate a phylogenetic tree that clusters by allele. Among these are absence of fixed difference between species, presence of shared neutral SNPs, and allelic associations among shared SNPs.

**Figure 2:**
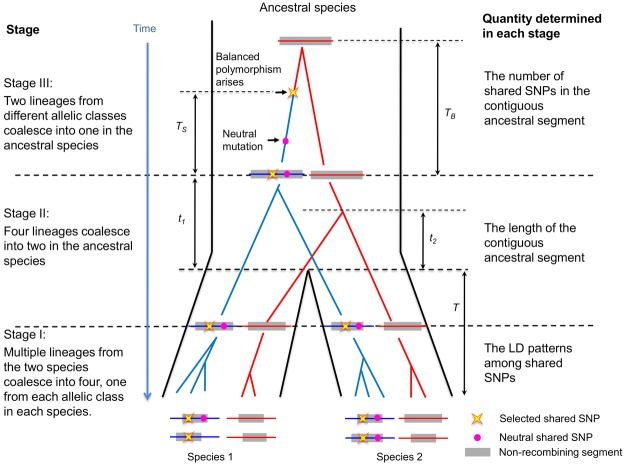
The genealogy of the contiguous ancestral segment. The proportion of each stage has been distorted for illustration purpose (e.g., stage I should be much shorter compared to stage II). See main text and Table1 for the meaning of symbols.

Motivated by these considerations, we ask (i) What is the *distribution of the length of the ancestral segment* for a sample of four chromosomes, one from each allelic class in each species? (ii) What is the *expected number of shared neutral polymorphisms* in the ancestral segment for that sample? (iii) What are the *expected number of shared neutral polymorphisms* and *the LD patterns* among them, for a sample of more than four chromosomes? The genealogy of the ancestral segment can be divided into three stages (Fig.2), the properties of which determine the answers to the three questions: in stage I, multiple lineages from the two species coalesce into only four lineages, one from each allelic class in each species; in stage II, the four lineages coalesce into two in the ancestral species, one lineage in each allelic class; in stage III, the remaining two lineages coalesce into one in the ancestral species.

### THE LENGTH OF THE ANCESTRAL SEGMENT

We first derive the length of the ancestral segment, which corresponds to the scale over which there may be shared neutral SNPs between species, but there cannot be fixed differences. The length is determined by the recombination events on the genealogy in stages I and II (Fig.2). We begin by considering the length of the ancestral segment for a sample of four chromosomes, one from each allelic class in each species (stage I does not exist in this case). In the Supporting Information, we show that so long as *T*>>*N_e_*, the effects of complex recombination events in the history of the sample can be neglected. In other words, the ancestral segment is approximately the segment in which no recombination event occurs until all the lineages from the same allelic class coalesce into their most recent common ancestor (MRCA).

The duration of stage II includes the split time (*T*) and the time (*t*) required for lineages carrying the same allele to coalesce in the ancestral species (Fig.2). Denoting the coalescent times for the two A1 lineages and the two A2s by *t*_1_ and *t*_2,_ respectively: 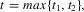


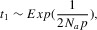


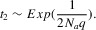


Therefore, the probability density of *t* is: 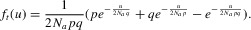
1

On each side of the selected site, the length of the ancestral segment is well approximated by the distance to the nearest crossover point in stage II. We denote this distance by *X* and the distance to the nearest crossover point in each lineage by 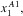 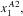 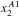, and 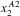, respectively, so 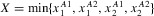. To calculate the distribution of *X*, we rely on the fact that 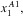 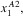 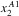, and 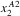 are all exponentially distributed: 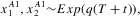


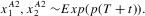


If we assume that 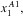 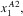 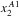, and 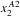 are independent of each other, the conditional distribution of *X* given *t* can therefore be approximated by: 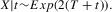
2

Equation 7 slightly underestimates *X*, because the two lineages that coalesce first share an ancestor in the time period between *t*_1_ and *t*_2_ and thus the reduction in the ancestral segment in that time period is overestimated by about a third. When *N_a_*<<*T*, that time period is negligibly short compared to the total length of stage II, so equation 7 performs well (as confirmed by simulations). For simplicity, we therefore rely on the approximation in equation 7 in what follows.

Combining equations 4 and 7, we obtain the probability density function of *X* as: 
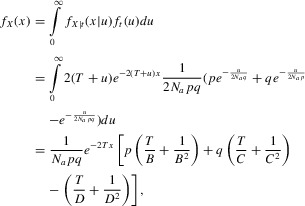
3where 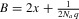, 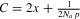, and 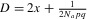.

When *p* = *q* = 0.5, equation 8 can be simplified to: 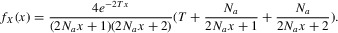


The distribution is insensitive to changes in *p* (Table S1), because the shape of the distribution is primarily determined by recombination events that occur before the species split (i.e., when there are two species), the rates of which do not depend on *p*. Using a similar approach, we also derive the length distribution of segments with the orange or yellow topologies (see Supporting Information).

Since recombination events on either side of the selected site are independent, the length of the ancestral segment is the sum of the lengths on both sides, and the distribution of the total length is given by the convolution of the one-sided distribution with itself. Considering parameters appropriate for humans and chimpanzees, we find that the predicted distribution of the length agrees well with the simulation results (Fig. S1, Table S1).

Our result for the length of the ancestral segment has important implications for genome scans of ancient balancing selection. Specifically, equation 8 shows that the length of the ancestral segment shrinks exponentially with *T*. For instance, assuming plausible parameters for humans and chimpanzees (*N_a_* = 50,000, *T* = 250,000, *P* = 0.5 and a recombination rate of *r* = 1.2 × 10^−8^ per bp per generation), the expected length of the ancestral segment on one side of the selected site is 131 base pairs (bp) and the 95% quantile is about 400 bps. In contrast, for *Drosophila melanogaster* and *D. simulans* (analyzed, e.g., in Langley et al. 2012; Bergland et al. 2013), the ancestral segment on each side of a single ancient balanced polymorphism will only be two base pairs on average and the upper 95% quantile is six base pairs (assuming *N_a_* = 10^6^, *T* = 2×10^7^, *P* = 0.5 and *r* = 1.2 × 10^−8^). This illustrates that the ancestral segment is extremely short for species with too old a split time. However, as we have discussed above, the split time needs to be sufficiently old for there to be little or no ILS by chance. Thus, *T* must be much greater than *N_e_*, but much smaller than 1/*r*. In this regard, humans and chimpanzees are a particularly well-suited pair, with *N_e_* ≈ 10^4^ << *T* ≈ 2.5 × 10^5^ << 1/*r* ≈ 8 × 10^7^.

### THE NUMBER OF SHARED SNPs IN THE ANCESTRAL SEGMENT

SNPs shared between species can be identical by descent or be generated by recurrent mutations. Therefore, observing a single shared SNP does not in itself provide compelling evidence for ancient balancing selection. Patterns of genetic variation in the ancestral segment, however, can provide more specific evidence for trans-species balanced polymorphism (Leffler et al. 2013). Notably, this segment may carry neutral polymorphisms shared identical by descent in addition to the selected one. The expected number of such shared neutral SNPs at a given genetic distance from the selected site is proportional to the coalescent time between the two lineages remaining in stage III (Fig.2). In what follows, we therefore derive expressions for this coalescent time.

We begin by considering the limit of an infinitely old balanced polymorphism. The coalescent process of two lineages at genetic distance *d* from the selected site can be described as a *finite-island model* with two subpopulations, corresponding to the two allelic backgrounds (Hudson and Kaplan 1988; Nagylaki 1998). Let *T_1_* be the coalescent time for two lineages carrying the A1 allele, *T_2_* for two lineages carrying the A2 allele, and *T_B_* for two lineages with different selected alleles. Conditioning on the outcome of the first step of the Markov process, we can write down recursive equations for the expected coalescent times: 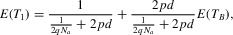


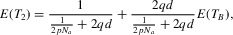


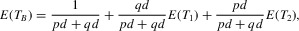
from which we obtain: 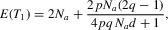


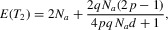


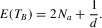


The results are similar to but slightly different from previous study (Kamau et al. 2007), because we assume that the two allelic classes have unequal frequencies and that there is no reverse mutation or allele turnover.

The expected number of shared neutral SNPs follows from the coalescent times. If the ancestral segment is *L* base pairs long (not including the selected site), with a uniform recombination rate *r* and a uniform mutation rate μ, the expected number of shared neutral SNPs is obtained by summing over distances: 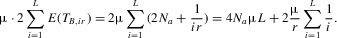


When the balanced polymorphism is not infinitely old, the coalescent process differs before and after the balanced polymorphism arose. We assume that the balanced polymorphism arose *T_S_* generation after the end of stage II (Fig.2), so the coalescent process at a neutral site *d* Morgans away from the selected site can be described as follows. In the first *T_S_* generations (the selection phase), the state of the sample of two lineages in generation *t* is described by (*n*_1_, *n*_2_), where *n*_1_ is the number of lineages carrying the derived allele A1 and *n*_2_ is the number of lineages carrying the ancestral allele A2. Once the two lineages coalesce, the allelic identity is no longer important, so we merge the two states (1,0) and (0,1) into one state, denoted by (*). Let *i* = 1, 2, 3, 4 refer to the four states (1,1), (0,2), (2,0), and (*), the transition matrix (*P_S_*) between them is: 
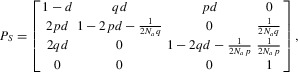
in which the element in the *i*th row and *j*th column is the probability of moving from state *i* to state *j* in one generation.

After the first *T_S_* generations (the neutral phase), when balancing selection no longer plays a role, there are two possible states, corresponding to two (**) and one lineage (*) remaining, and the transition matrix between these two states is: 
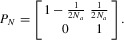


The transition matrix from states (1,1) (0,2), (2,0), and (*) in the neutral phase to states (**) and (*) in the neutral phase is: 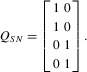


The transition from (2,0) at the end of the selection phase to (*) at the beginning of the neutral phase comes from our assumption (forward in time) that the derived selected allele goes to frequency *p* immediately after it arises, so backwards in time, any two lineages carrying the derived allele have to coalesce at generation *T_S_*.

The average coalescent time of interest is equal to the expected number of steps needed to enter state (*) starting from state (1,1), which can be calculated numerically using the transition matrices (see Supporting Information).

Alternatively, a closed-form approximation of the average coalescent time can be obtained by ignoring recombination once the process leaves state (1,1). This approximation will lead to an underestimate of the coalescent time; however, it is expected to perform well, because the ancestral segment is so short that the recombination rate within it is on the order of 1/(2*T*), which is much smaller than the coalescence rate of approximately 1/(2*N_a_*) when *N_a_*<<*T*. In this approximation, the transition matrix during the selection phase *P_S_* simplifies to an upper triangular matrix: 
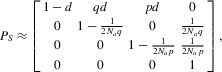
from which we can obtain an analytic approximation of the expected coalescent time *E*(*T_B_*) (see Supporting Information).

**Figure 3:**
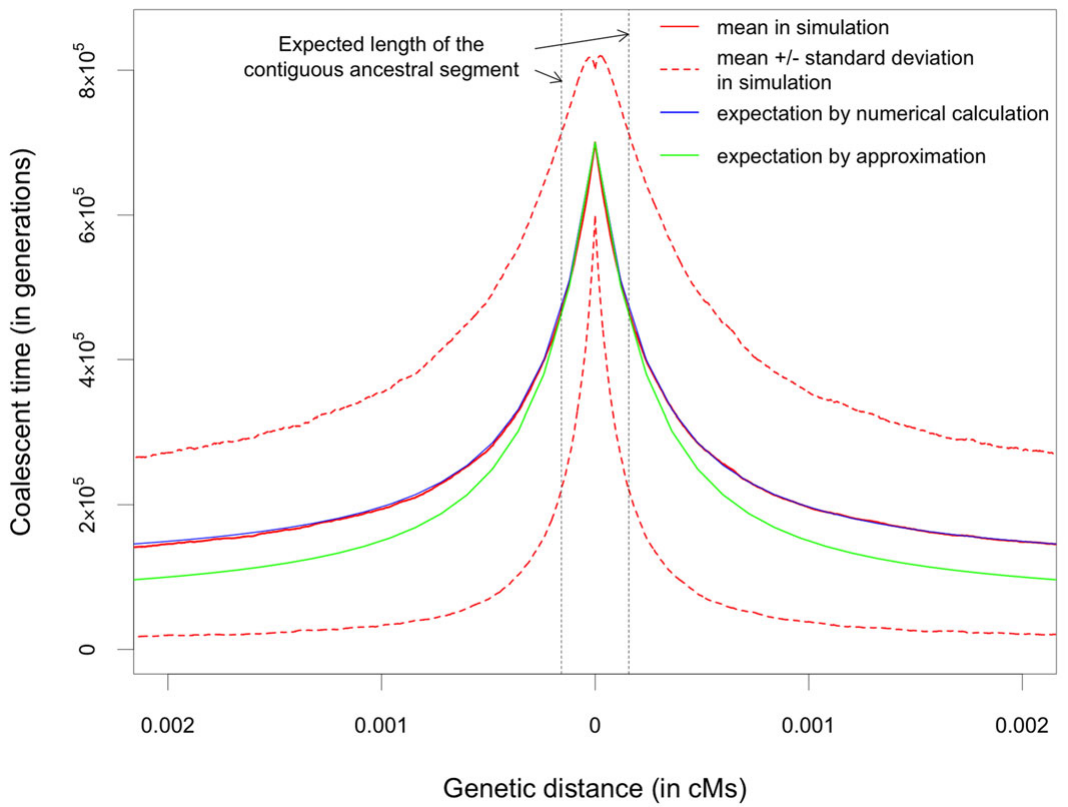
Expected coalescent time between two lineages carrying different alleles. Parameters are chosen to be plausible for the ancestral population for humans and chimpanzees: *N_a_* = 50,000, *p* = 0.5, and *T_S_* = 600,000 generations (see Supporting Information). The mean and standard deviation of the simulation results were obtained from 10,000 replicates.

In Figure3, we compare the expected *T_B_* obtained from the numerical calculation, analytic approximation, and coalescent simulations. We consider parameters that are plausible for the ancestral species of humans and chimpanzees. As expected, the numerical calculation predicts the mean coalescent time from simulations very well, whereas the analytic approximation tends to slightly underestimate the mean. However, the approximation is much faster and easier to obtain than numerical calculation, and it gives similar results on the scale of the ancestral segment.

**Figure 4:**
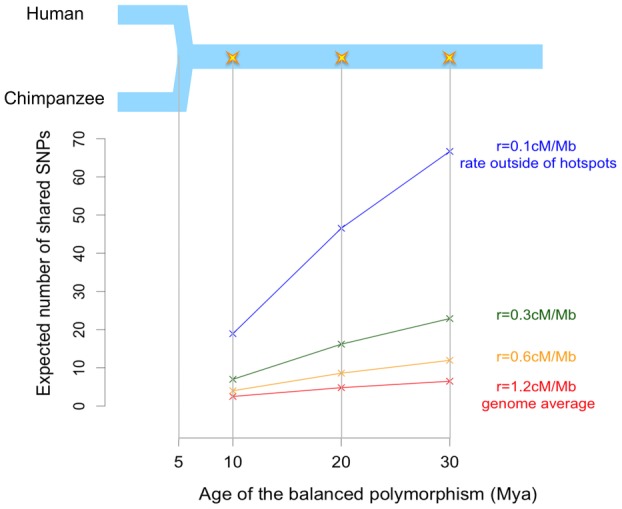
Expected number of shared SNPs in the contiguous ancestral segment. The upper panel is a schematic diagram of the demographic history of humans and chimpanzees, which merged into an ancestral species five million years ago (Mya). The star indicates the age of the balanced polymorphism. The lower panel shows the expected number of shared SNPs (including the selected one) in the two-sided contiguous ancestral segment. Note that the age of the balanced polymorphism here is measured from present, which is the sum of *T_S_* and the lengths of stages I and II.

Based on these results, we can predict the expected number of shared neutral SNPs around a trans-species balanced polymorphism in humans and chimpanzees (Fig.4). As an illustration, assuming the average mutation and recombination rates for humans, when the balanced polymorphism is 20 million years (Myr) old from present, we expect about two additional shared neutral SNPs—more if the polymorphism is older. For a region where recombination is lower than the genome average, the ancestral segment is longer and could plausibly contain as many as a dozen shared neutral SNPs. In turn, for *D. melanogaster* and *D. simulans*, even though the contiguous ancestral segment is expected to be only two base pairs long, both sites can harbor shared SNPs if the balanced polymorphism is sufficiently old (assuming that the balanced polymorphism is infinitely old and the ratio of mutation rate to recombination rate is one). These results indicate that, despite the short ancestral segment, there may be a detectable signal from shared neutral SNPs within it. Moreover, assuming that selection acts on a single site, the targets will be very well delimited, yielding only a handful of possible SNPs to followup with functional assays.

### LD PATTERNS OF SHARED SNPs IN A LARGER SAMPLE

Given that in genome scans for selection, the targets are not known a priori, it is helpful to consider what to expect in a sample of more than four chromosomes, where we do not condition on observing the balanced polymorphism. If *n* chromosomes are sampled at random from each species, the probability of capturing both alleles at the selected site in both species is (1 − *p^n^* − *q^n^*)^2^. This probability obviously increases with sample size and is maximized at *p* = *q* = 0.5 for any given sample size.

Assuming that the trans-species polymorphism is observed in the sample, the number of shared neutral SNPs is expected to increase slightly with sample size. For a sample of more than four chromosomes, the *ancestral segment* can be defined as the distance at which at least one lineage from each allelic class in each species experiences no recombination in stages I and II. This is the segment in which shared neutral SNPs can be found, and its expected length will increase slightly with sample size, because larger samples are less sensitive to chance recombination events very close to the selected site during stage I. However, because stage I is much shorter than stage II (on average approximately 4*N_e_* generations compared to approximately *T* generations), this effect will be negligible for the parameters considered (Table S2).

Increasing the sample size also affects the LD levels between the shared neutral and selected SNPs. If a shared neutral SNP is present in a sample of four chromosomes (one from each allelic class in each species) in addition to the selected SNP, the two shared SNPs must be in perfect LD in both species (i.e., there will be only two haplotypes and *r*^2^ = 1). This is not the case for larger sample sizes, as becomes clear if we consider the ancestral segment as two parts. In the part that is adjacent to the selected site, no recombination occurs in any lineage in either stage I or II, and thus the phylogenetic tree topology is exactly the same as for the selected site. Therefore, any shared neutral SNPs (that arise from mutations in stage III) will be in perfect LD with the selected one, as is the case for the entire ancestral segment for a sample of four chromosomes. The other part of the ancestral segment is distal from the selected site and defined by there being recombination in some lineages in stage I but no recombination in state II. The genealogy of the sample in this segment does not cluster by species, nor does it cluster entirely according to the selected allele. Thus, shared neutral SNPs in this part are in imperfect LD with the selected SNP (i.e., there are more than two haplotypes). In general, increasing the sample size has a slight influence on the total length of the ancestral segment and moves the boundary between the two parts closer to the selected site, leading to a lower fraction of shared neutral SNPs in perfect LD with the selected one. Therefore, increasing the sample size will reduce the expected LD between the selected site and shared neutral SNPs at any given genetic distance.

The level of LD between the selected site and a shared neutral SNP at a distance of *d* Morgans can be thought of as follows. For the neutral site to segregate in both species, it must have arisen in the ancestral population and remain polymorphic in both species in stages I and II. Therefore, when focusing on one species, at the end of stage I (when there are two lineages, one in each allelic class), one allele (B1) at the neutral site must be linked to A1 and another allele (B2) linked to A2. It follows that whether an A1 lineage at present carries allele B1 or B2 depends on whether it remained in the A1 class or migrated to the A2 class during stage I. Therefore, the LD between the selected and the shared neutral SNPs under consideration reflects the correlation between the allelic identities of the lineages at the beginning and the end of stage I.

**Figure 5:**
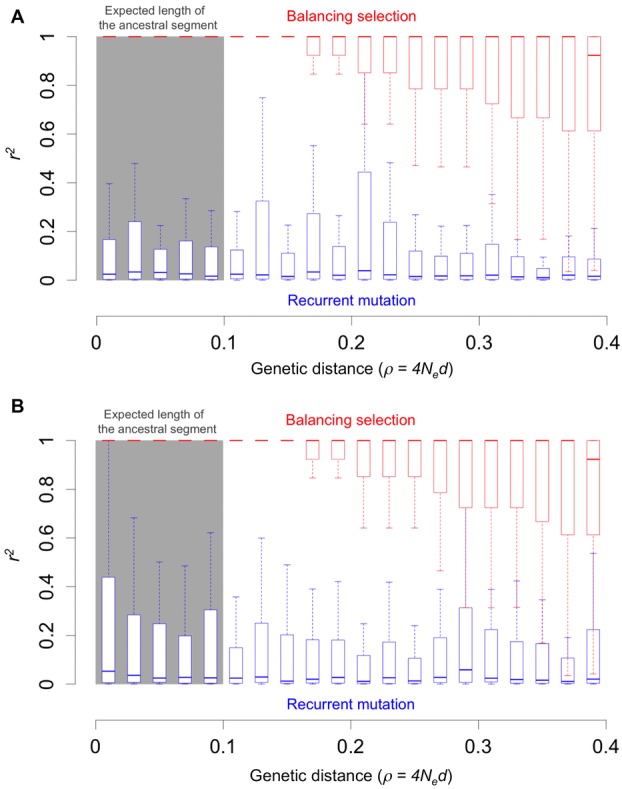
LD between shared SNPs generated by balancing selection or by recurrent mutations. Fifty chromosomes were sampled from each species in both scenarios. We assumed an allele frequency of 0.5 for the scenario of balancing selection and sampled an equal number of chromosomes from each allelic class (see Supporting Information for details of the simulations). Panels (A) and (B) show the distribution of *r^2^* in the two species, respectively.

This correlation can be thought of in terms of the probability that a given lineage does not switch allelic identity during stage I; we denote this probability for a sample of *n* lineages carrying the same allele by *R_n_*. In the SI, we derive an approximation for *R_n_* and show how it relates to commonly used summaries of pairwise LD. Simulations show that this approximation underestimates the extent of LD, but nonetheless shows that the LD levels between the selected and neutral sites are expected to be high even in a large sample (Figs. S2 and S3). In conclusion, if a shared neutral SNP is observed in a sample of more than four chromosomes, it is expected to be in strong but not necessarily perfect LD with the selected site (Fig.5).

### FLUCTUATION IN ALLELE FREQUENCY OF THE BALANCED POLYMORPHISM

Our results are derived under the assumption of constant allele frequency of the balanced polymorphism, but, in reality, the allele frequencies will fluctuate, both due to genetic drift and to shifts in selection pressures. Conditional on the balanced polymorphism not being lost in both species, we expect our results to hold so long as the selected allele frequencies fluctuate around a long-term mean value rapidly (relative to *N_e_* generations). Coalescent simulations of overdominance allowing for fluctuating allele frequencies confirm this intuition (see Supporting Information). The same reasoning suggests that our results will hold for other mechanisms of balancing selection, given that models of changing environments or negative frequency-dependent selection usually lead to oscillations of the allele frequencies on a much faster time scale (tens of generations, e.g., Stahl et al. 1999; Tellier and Brown 2007) than does the overdominance model that we simulated (where it is on the order of *N_e_* generations, see Fig. S4A).

### SIMULATIONS OF NEUTRAL RECURRENT MUTATIONS

To provide clear-cut evidence for ancient balancing selection, the signals associated with shared SNPs need to be highly unlikely to occur under neutrality. One way that shared SNPs could arise under neutrality is by ILS. As noted, when *T* is much greater than *N_e_*, the probability of neutral trans-species polymorphisms is negligible. Moreover, fluctuations in population size will, if anything, tend to *decrease* the probability of neutral trans-species polymorphisms, because bottlenecks would increase the chance of neutral polymorphisms being lost.

Another way that shared SNPs could arise is by recurrent mutation, that is, be identical only by state (Asthana et al. 2005; Hodgkinson et al. 2009). To distinguish trans-species SNPs from those shared identical by state, we have to consider their footprints at nearby sites. Notably, while trans-species polymorphisms may be accompanied by shared neutral SNP(s) in strong LD, such signals are highly unlikely to be generated by neutral recurrent mutations alone.

As an example, we consider a model of recurrent mutations for humans and Western chimpanzees, incorporating differences in mutation rates between CpG and non-CpG sites (as in Leffler et al. 2013). We simulated diversity patterns in 10,000 replicates of 100 kb segments (together equivalent to one third of the human genome of 3 × 10^9^ bps) under two demographic scenarios: one with constant effective population sizes and the other with bottlenecks in both species (see Supporting Information and Table S4 for details). Under the first scenario, only 2% of the segments contain shared SNP pairs within 400 bps and only 7% of these pairs are in significant LD with the same alleles in coupling in both species (at the 5% level, using χ^2^**-**test). Moreover, no replicate contains more than one pair of shared SNPs within 400 bps. As expected, the demographic scenario with bottlenecks (Li and Durbin 2011; Prado-Martinez et al. 2013) leads to even fewer shared SNP pairs (results not shown).

In addition, LD patterns among shared SNPs differ for recurrent mutations and under a balanced trans-species polymorphism (Fig.5). In the balancing selection case, *r^2^* between the selected and neutral sites is close to one within the expected length of the ancestral segment and decreases with genetic distance, as expected from our analytic results. In contrast, under recurrent mutations, the *r^2^* between shared SNPs within the same range is usually quite low regardless of the genetic distance (on average around 0.1 for a sample of 50 chromosomes). The reason for such LD pattern is that, at such close distances, the two sites almost always share the same genealogies, and the LD between them depends on the branches on which mutations at each site arose.

## Discussion

### INTERPRETING EXISTING EXAMPLES

Our modeling helps to interpret cases of trans-species polymorphisms found to date, and design additional genome-wide scans. For example, a recent study showed that the A/B polymorphism underlying the ABO blood groups is shared between humans and gibbons identical by descent, indicating that the polymorphism originated before the species split approximately 19 million years ago (Ségurel et al. 2012). Strong evidence for identity by descent includes the lack of fixed difference between species and the presence of a second shared (nonsynonymous) SNP between humans and gibbons about 100 bps away from the A/B polymorphism, which is in strong LD with the same alleles coupled in both species. This pattern of shared SNPs is consistent with our expectations: at the genome average recombination rate, an ancestral segment shared between humans and gibbons has an expected length of 35 bps on each side of the selected site, with an 95% quantile of 150 bps. Therefore, this example conforms with the footprints that are expected to be highly specific to a trans-species balanced polymorphism.

A number of additional targets of ancient balancing selection were recently reported in a study of humans and chimpanzees (Leffler et al. 2013). In a handful of cases, the diversity levels in humans are comparable with the genome average divergence between humans and chimpanzees (also see Rasmussen et al. 2014); furthermore, the phylogenetic trees around those shared SNPs had the orange topology, providing compelling evidence for trans-species polymorphisms. Among them, two cases exhibited polymorphism patterns indicative of more complex scenarios of trans-species balanced polymorphisms (Table2). A noncoding region near gene *FREM3* is much longer than expected given the average recombination rate in human genome. One simple explanation is that the recombination rate in this region has been low in both lineages since the split between species, consistent with the low estimated recombination rate in this region (Frazer et al. 2007). An alternative is that recombination between the two haplotypes is selected against, as might be the case if there are two or more sites under selection, with epistasis among them (Kelly and Wade 2000). The large number of shared SNPs in this region further suggests that the balanced polymorphism is old. Accordingly, three of these shared SNPs are also segregating among gorillas and orangutans, the latter of which split from humans at least 11 Myr ago (Prado-Martinez et al. 2013).

**Table 1:** List of parameters

Symbols	Definitions
*N_a_*, *N_e_*	Effective population sizes for the ancestral and descendant species, respectively
*p*, *q* = 1 *– p*	Equilibrium frequencies for allele A1 and A2 at the biallelic polymorphism under balancing selection
*T*	Species split time in generations
*r*	Recombination rate per base pair per generation
μ	Mutation rate per base pair per generation
*X*	Length of the one-sided ancestral segment in Morgans
*d*	Genetic distance between a neutral site and the selected site, in Morgans
*T_S_*	Age of the balanced polymorphism in generations
*P_S_*, *P_N_*, *Q_SN_*	Transition matrices in the selection phase, in the neutral phase and between the two phases, respectively
*s*	The harmonic mean of the selection coefficients of the two homozygotes in an overdominance model

**Table 2:** Two regions with trans-species polymorphisms shared between humans and chimpanzees

		Expected length of the		Number of shared	
Region (the nearest gene)	Observed length of the region^a^, ^b^	ancestral segment (upper 95% quantile)	Number of all shared SNPs (number of shared SNPs at non-CpG sites)^b^	SNPs in other primate speciecs^c^	LD pattern among shared SNPs
*FREM3*	6.7 kb	263 bp	13	3	In almost perfect LD
		(800 bp)	(13)	(Among both 31 gorillas	with the same alleles
				and 10 orangutans)	coupled in both species
*IGFBP7*	870 bp	263 bp	5	1	**Within cluster 1 or 2:** in
(clusters 1 and 2)		(800 bp)	(2)	(Among both 31 gorillas	almost perfect LD with
				and 10 orangutans)	the same alleles coupled
					in both species
					**Between clusters 1 and 2:** in strong LD with the opposite phases (i.e., with different alleles coupled) in the two species
*IGFBP7*	200 bp	263 bp	3	None	**Within cluster 3:** in almost
(cluster 3)		(800 bp)	(1)		perfect LD with the same
					alleles coupled in both species
					**With clusters 1 and 2:** in strong LD in chimpanzees but not in LD in humans

a The span of the region is defined as the distance between the two outermost shared SNPs that are in significant LD in both species.

b Data are from Leffler et al. (2013).

c Data are from Great Ape Genome Project (Prado-Martinez et al. 2013).

A second case is a noncoding region in the first intron of the gene *IGFBP7*, which contains eight shared SNPs in three LD clusters (Table2). The first two clusters are in almost perfect LD, but involve a switch in phase between the two species (i.e., with different alleles coupled in the two species), which is also seen at *ABO* (Ségurel et al. 2012). This pattern is unlikely to be due to recurrent mutations on the genealogy of a trans-species polymorphism (Fig. S5A), as at least two mutations would be required; more likely are complex recombination events in stage II (Fig. S5B). Another pattern of interest is the existence of a third LD cluster (approximately 600 bps away from the first two clusters), which suggests that distinct balanced polymorphisms led to the first two and the third clusters, with little or no epistasis between them. Moreover, there are two diversity peaks in the region: one underlying clusters one and two and the other underlying cluster three. The high density of shared SNPs in the first two clusters again suggests the balanced polymorphisms might be old, which is supported by the fact that one SNP in cluster one is also found to be shared by other primate species (Prado-Martinez et al. 2013). Overall, among the six strongest candidate regions, the number of shared SNPs is predictive of whether SNPs are shared with other species, consistent with our expectation that older balanced polymorphism should lead to a higher density of shared neutral SNPs. These examples illustrate that our results can also aid understanding of more complicated cases of balancing selection than those considered in our simple model.

### CONSIDERATIONS FOR FUTURE STUDIES OF TRANS-SPECIES POLYMORPHISMS

Our analyses highlight the split time, *T*, as a key parameter determining the suitability of a pair of species. *T* needs to be much larger than *N_e_* to avoid being swamped by false positives due to ILS. For a simple demographic model with random mating and constant population sizes, the probability of neutral trans-species polymorphism is on the order of *e^−T/Ne^*, which is negligible when *T/N_e_* is sufficiently large.

This probability is affected by demography, however. While population bottlenecks will decrease this probability, population structure and admixture increase the mean and variance of the neutral coalescent time, and thus could make neutral trans-species polymorphism more likely. Recent demographic events should have relatively little effect, however. In humans, in particular, when the gene flow from Denisovans and Neanderthals into modern humans is considered, the probability of trans-species neutral polymorphism between humans and chimpanzees for those introgressed regions will increase by only about threefold (assuming *N_e_* = 10,000, a generation time of 20 years and *T* = 440 Kya between modern humans and these archaic humans; Green et al. 2010). Thus, even with such archaic admixture into modern humans, ILS with chimpanzees is highly unlikely by chance.

While *T/N_e_* needs to be sufficiently large, *T* needs to be smaller than 1/*r*, or the ancestral segment will be too short to contain enough information that distinguishes between balancing selection and neutral, recurrent mutations. It follows that species with large effective population sizes are less appropriate. As we have shown, for *D. simulans* and *D. melanogaster*, and more generally for species with *N_e_* on the order of 10^6^ and recombination rate on the order of 10^−8^ per bp per generation (Comeron et al. 2012), the ancestral segment around a trans-species polymorphism is expected to be only a few base pairs long. In practice, even if there were additional shared SNPs near the selected one, it would be hard to align sequencing reads with several polymorphic sites in close proximity, and the trans-species polymorphism might be missed.

In contrast, mammalian species have relatively small *N_e_* (Leffler et al. 2012), so some of mammalian species pairs should be well suited for scans for trans-species polymorphisms. In addition to humans and chimpanzees, another example of a suitable species pair is rhesus macaques and baboons that have similar demographic parameters, that is, *T* (Perelman et al. 2011) and *N_e_* (Perry et al. 2012). Other examples of suitable pairs of species include *Drosophila* species with relatively low genetic diversity levels, for example, *D. miranda* and *D. bogotana*. These two species are narrow endemics with distinct habitats, and show reproductive isolation between them. The divergence between the two species (approximately 5%) is estimated to be an order of magnitude higher than their diversity levels (0.62% for *D. bogotana* and 0.36–0.53% for *D. miranda*; Machado et al. 2002; Bachtrog and Andolfatto 2006), which suggests that neutral trans-species polymorphisms are unlikely and that the footprint of balanced polymorphism should be sufficiently long to be detectable.

### EFFECTS OF DEPARTURES FROM MODEL ASSUMPTIONS

Our analyses rely on several simplifying assumptions about the selection dynamics and the recombination and mutation processes, violations of which would render our quantitative results inaccurate. Notably, we assume that selection acts on a single-locus biallelic polymorphism that arose after the establishment of selection pressure. However, balancing selection may act on standing variation. If the selection pressure was established after the polymorphism arose, we need to substitute the age of the balanced polymorphism *T_S_* by the age of the selection pressure when calculating the expected number of shared SNPs, and the approximation should still hold.

Also in violation of our assumptions, several known examples of trans-species polymorphism (e.g., MHC, S-locus in plans) exhibit a large number of functionally different alleles that usually have a large target size and involve strong epistasis. In this case, although the selection dynamics clearly deviate from our model assumptions, empirical evidence suggests that multiallelic trans-species polymorphisms may lead to unexpectedly large numbers of shared SNPs (Ioerger et al. 1990; Charlesworth et al. 2006). In this scenario, the LD levels among shared SNPs are not necessarily as strong as what predicted in the biallelic model, but genome-wide scans for shared SNPs in significant LD are still likely to capture some of these cases.

We also made some simplifying assumptions about the recombination process. By assuming a constant recombination rate per base pair across sites and over time, we ignored, in apes, for example, the potential effects of hotspots and their rapid evolution (Myers et al. 2010). However, given that the time scale for trans-species polymorphism is so long and that genetic landscape exhibits rapid turnover on fine scale but appears fairly constant on large scale (of 1 Mb), it is plausible that the average recombination rate over megabase scale at present could reflect the long-term average recombination rate for any small region within it; if so, our approximation for the length of the ancestral segment should still hold. Another aspect of recombination that we ignored is gene conversion. We expect gene conversion to obscure the footprints of trans-species polymorphism, because it decreases the number of shared SNPs and leads to fixed difference between species when taking place in stage II, and it tends to reduce the LD among shared SNPs when occurring in stage I. Therefore, the method proposed in this article will have substantially lower power in regions of frequent gene conversion.

In addition, we assumed no subsequent mutation at the selected site after the first emergence of the balanced polymorphism, thus excluding the possibility of reverse mutation or allelic turnover at the selected site. Although this assumption is oversimplified, we expect it to be appropriate for balanced polymorphisms for which the selected target sizes are small or the mutation rate is low (Ségurel et al. 2012).

Moreover, neutral recurrent mutations on the genealogy of a trans-species polymorphism are not considered in our model. In stage II, occurrences of the same mutations on the two lineages in the same species lead to fixed differences between species; on the other hand, independent recurrent mutations in the two species give rise to shared SNPs that are identical by state in addition to those identical by descent. Therefore, additional shared SNPs can either strengthen or blur the signals of trans-species polymorphism, depending on the LD between them and the selected SNP (Wiuf et al. 2004). Since both gene conversion and recurrent mutations can lead to fixed differences between species in the vicinity of an ancient balanced polymorphism, less weight should be given to absence of fixed differences than presence of additional shared SNPs in high LD; nonetheless, when observed, the former phenomenon is an additional line of evidence in support for the presence of a trans-species polymorphism.

Finally, we note that although our approximations provide a good description of genetic variation patterns around a trans-species balanced polymorphism, they will not apply to cases of ancient balancing selection where the ancestral polymorphism was lost in one of the species. The detection of such cases will instead require consideration of other features of genetic variation data (DeGiorgio et al. 2014).

In summary, for appropriate pairs of species, genome-wide scans for trans-species polymorphism using these footprints should have reasonably high power (see Table S5) and extremely low false-positive rates, even when considering a wider set of assumptions than explicitly modeled. We emphasize, however, that this does not imply the FDR would be low, because the FDR depends on the ratio of the number of regions truly under balancing selection to the number of neutral regions in which these footprints are observed. Thus, even if these footprints are highly specific, if long-lasting balancing selection is very rare, true targets may be only a small proportion of candidate regions (Leffler et al. 2013).

### FUTURE DIRECTIONS

In this article, we focus on the ancestral segment that has the same genealogical structure as the trans-species balanced polymorphism. However, sites outside the ancestral segment also carry signals informative of trans-species polymorphism. In particular, Figure1 reveals a specific succession of topologies surrounding a trans-species polymorphism: the ancestral segment is always flanked by regions with yellow genealogical topology, an intermediate structure between allele-clustering (orange) and species-clustering (green and blue) patterns. By contrast, the flanking sequences of a shared SNP generated by recurrent mutations rarely have the yellow genealogical structure. This observation suggests that methods that explicitly infer underlying genealogical structure from DNA sequence data could leverage additional information about trans-specificity. Recently, several modeling frameworks have been developed for statistical inference of local gene genealogies (Li and Stephens 2003; McVean and Cardin 2005; Paul and Song 2010; Rasmussen et al. 2014). Such methods could potentially be modified to detect trans-species polymorphisms and to identify additional targets of ancient balancing selection. Since the method considered in this article has high power to detect trans-species polymorphisms with hitchhiking shared neutral SNPs, we expect these extensions to be most useful for cases where there is no shared neutral SNP nearby or the ancestral segment is short, but the flanking genealogies are highly informative.

## Supporting Information

Disclaimer: Supplementary materials have been peer-reviewed but not copyedited.

**Figure S1**. Distribution of the length of ancestral segment according to our approximation and in simulations. **Figure S2**. *R_n_* calculated from our approximation and obtained by simulation. **Figure S3**. Expected *r_2_* calculated from our approximation and obtained by simulation. **Figure S4**. The impact of fluctuations in the selected allele frequencies on the three summary statistics considered. **Figure S5**. Two scenarios that can generate shared polymorphisms in LD but with the opposite phases in the two species. **Table S1**. Summaries of the length of the contiguous ancestral segment **Table S2**. Influence of sample size on the number of shared neutral SNPs and LD among them. **Table S3**. Proportion of ancestral balanced polymorphisms that persist in both species until present **Table S4**. Parameter values used in simulations of neutral recurrent mutations. **Table S5**. Percentage of trans-species balanced polymorphisms accompanied by neutral shared SNP(s) in coalescent simulations
