# Supplementary material for: Footprints of ancient-balanced polymorphisms in genetic variation data from closely related species
Source: Evolution. 2015 Jan 16;69(2):431–46. doi: 10.1111/evo.12567 (PMC4335603; doi:10.1111/evo.12567)
Supplement: Supplementary file 1 — Figure S1. Distribution of the length of ancestral segment according to our approximation and in simulations. Figure S2. Rn calculated from our approximation and obtained by simulation. Figure S3. Expected r2 calculated from our approximation and obtained by simulation. Figure S4. The impact of fluctuations in the selected allele frequencies on the three summary statistics considered. Figure S5. Two scenarios that can generate shared polymorphisms in LD but with the opposite phases in the two species. Table S1. Summaries of the length of the contiguous ancestral segment Table S2. Influence of sample size on the number of shared neutral SNPs and LD among them. Table S3. Proportion of ancestral balanced polymorphisms that persist in both species until present Table S4. Parameter values used in simulations of neutral recurrent mutations. Table S5. Percentage of trans-species balanced polymorphisms accompanied by neutral shared SNP(s) in coalescent simulations [file evo0069-0431-sd1.pdf]

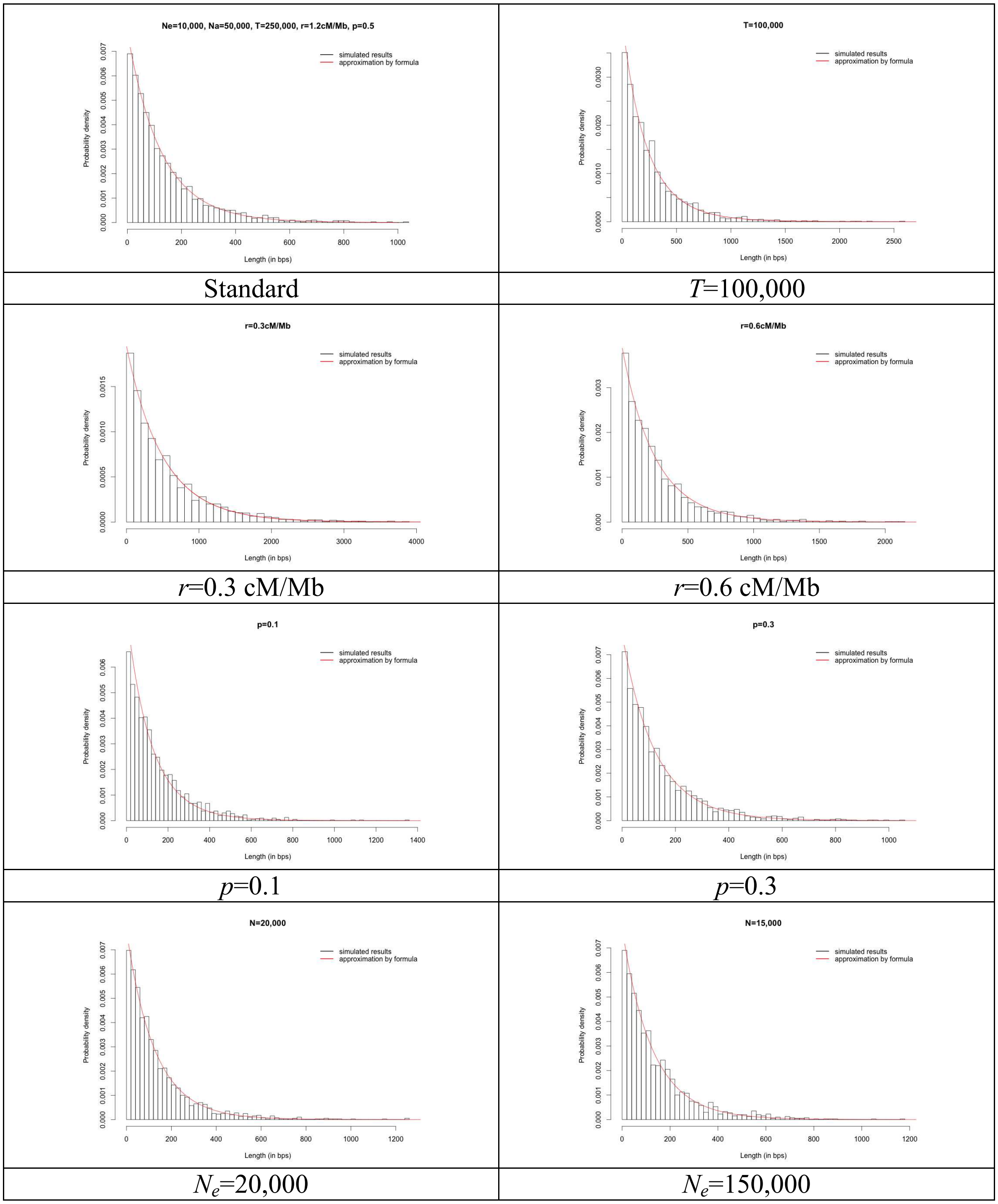


**Figure S1**: Distribution of the length of ancestral segment according to our approximation and in simulations. Unless otherwise specified, parameters were: $N_e$=10,000, $N_a$=50,000, $T$=250,000, $r$=1.2cM/Mb and $p$=0.5, which were chosen to be plausible for humans and chimpanzees. For all combinations of parameters tested, the distribution predicted by the approximation fits the simulation results very well.

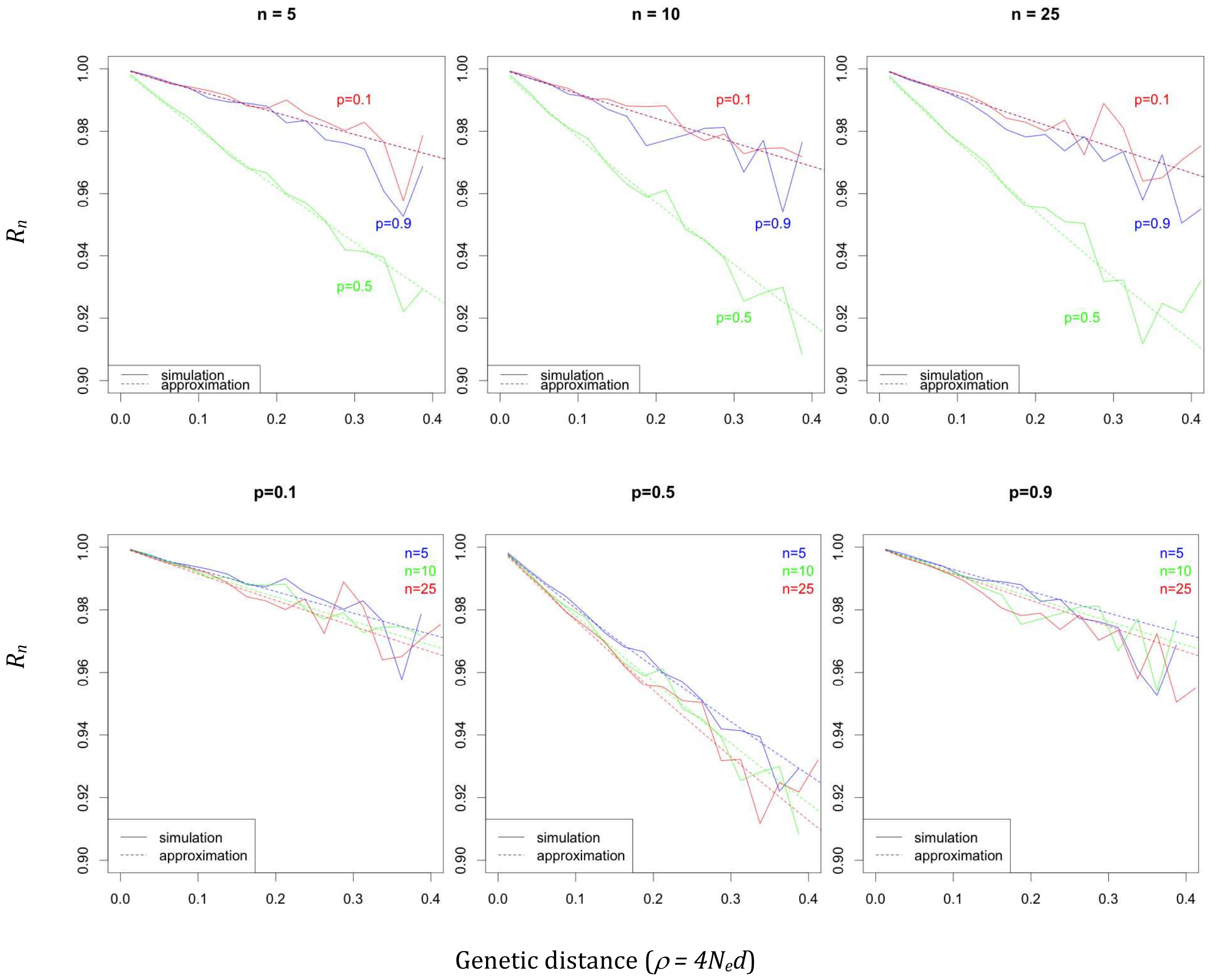


**Figure S2:** $R_n$ calculated from our approximation and obtained by simulation. The simulated segment is divided into bins of $6.25\times10^{-5}$ cM, and the mean $R_n$ is calculated from all SNPs in each bin. Only bins with more than 50 data points are shown. See Supplementary Methods for details of the simulations.

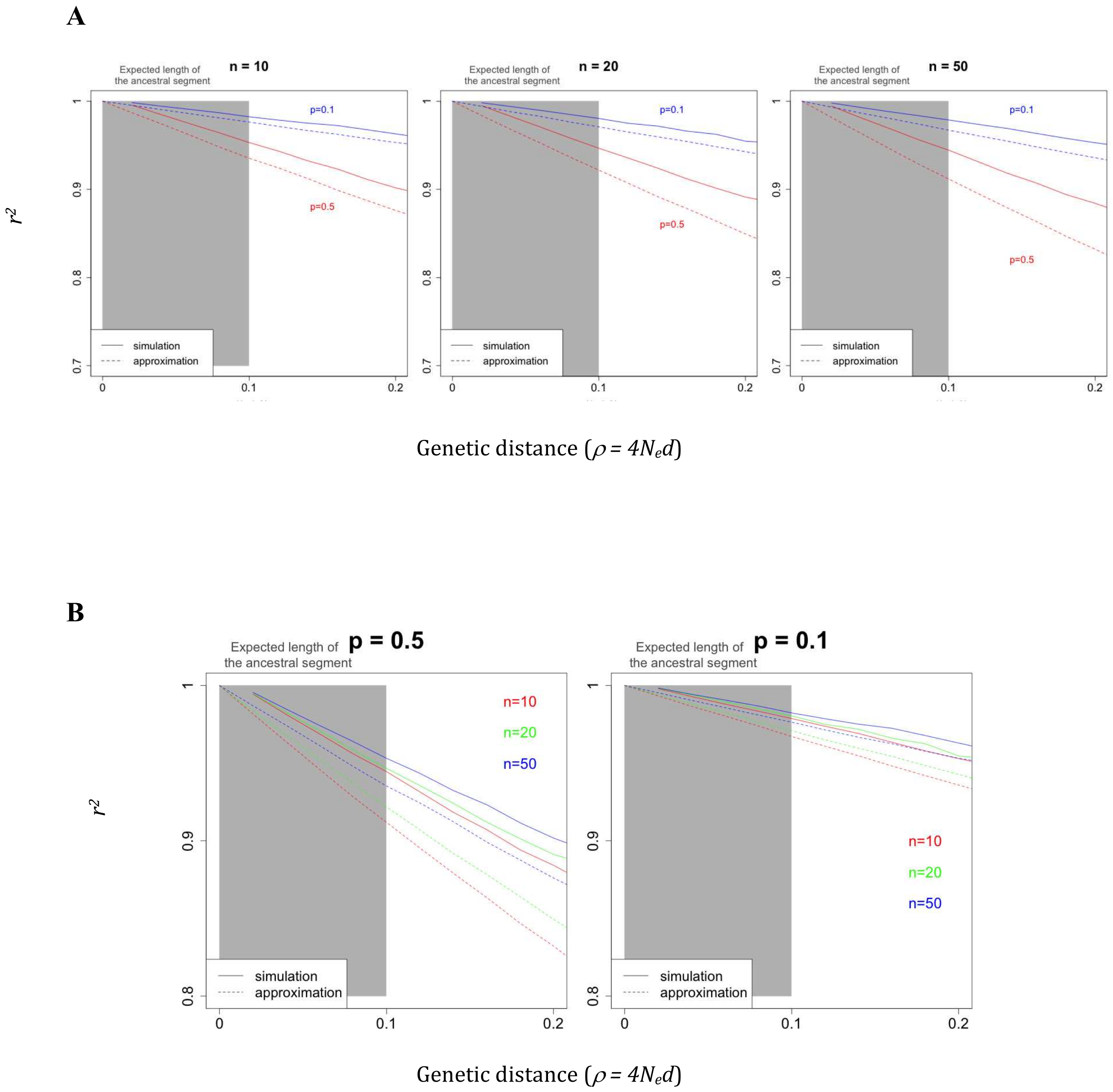


**Figure S3:** Expected $r^2$ calculated from our approximation and obtained by simulation. The simulated segment is divided into bins of $6.25\times10^{-5}$ cM and the mean $R_n$ is calculated from all SNPs in each bin. The expected $r^2$ from $R_n$ was calculated from 10,000 binomial sampling simulations as described in the supplementary Methods. (A) The mean $r^2$ decreases with sample size. (B) The mean (or expected) $r^2$ decreases with the product of the equilibrium frequencies of the two alleles (*pq*).

A

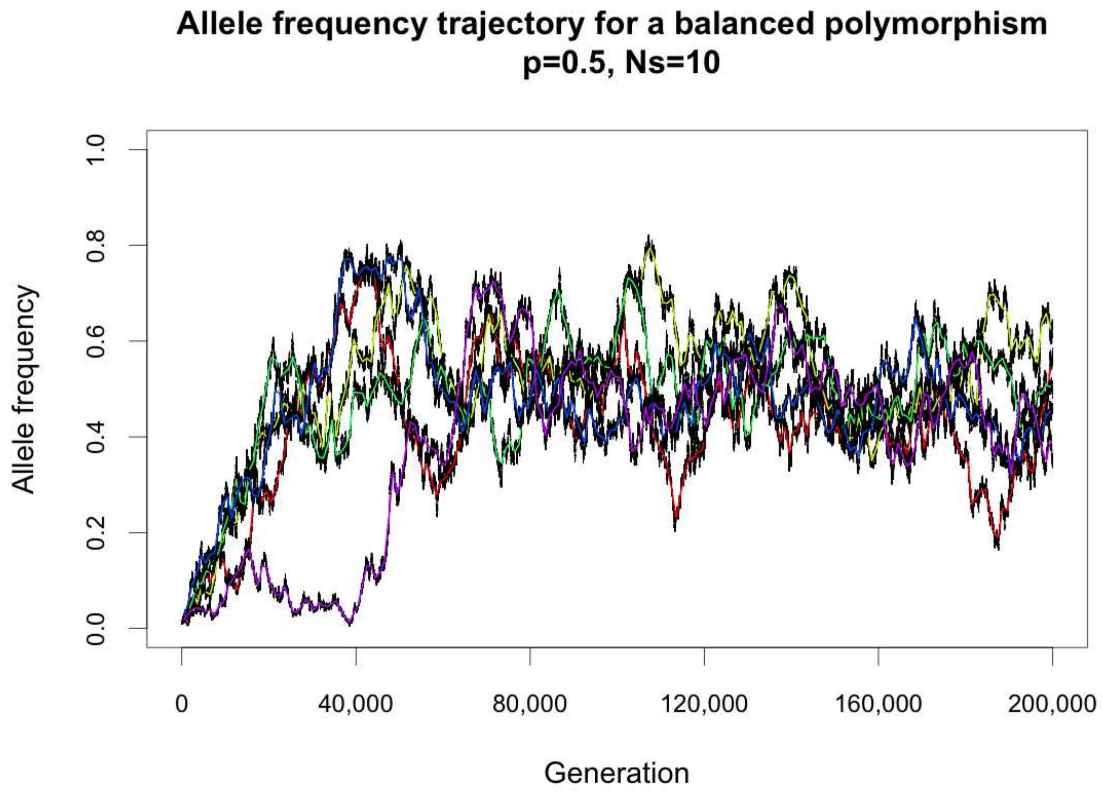
Allele frequency trajectory for a balanced polymorphism
p=0.5, Ns=10
Allele frequency
0.0
0.2
0.4
0.6
0.8
1.0
0
40,000
80,000
120,000
160,000
200,000
Generation

B

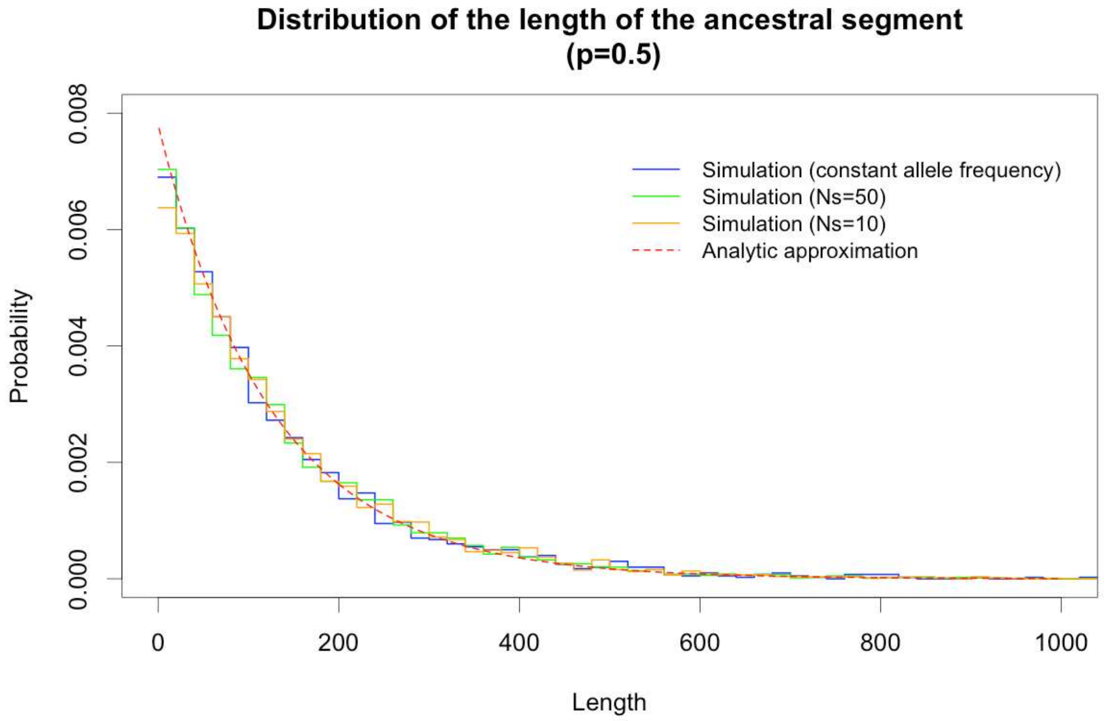
Distribution of the length of the ancestral segment
(p=0.5)
Probability
0.000
0.002
0.004
0.006
0.008
Simulation (constant allele frequency)
Simulation (Ns=50)
Simulation (Ns=10)
Analytic approximation
0
200
400
600
800
1000
Length

C

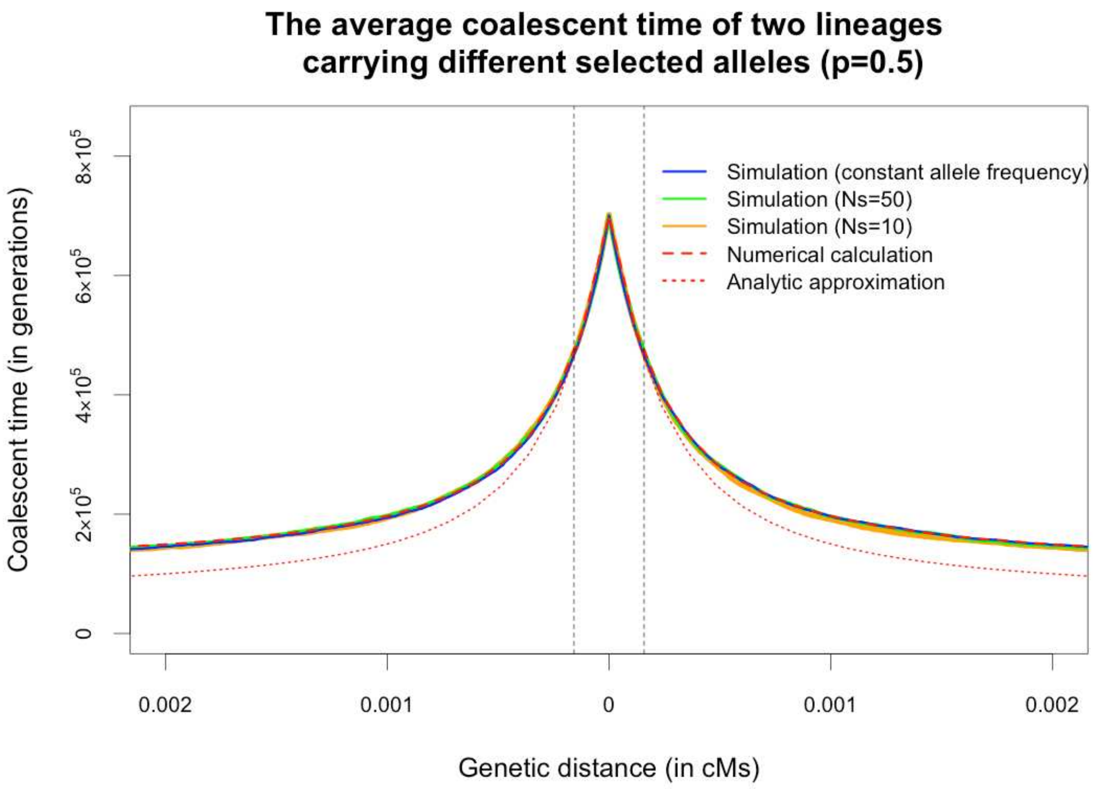
The average coalescent time of two lineages carrying different selected alleles (p=0.5)
Simulation (constant allele frequency)
Simulation (Ns=50)
Simulation (Ns=10)
Numerical calculation
Analytic approximation
Coalescent time (in generations)
Genetic distance (in cMs)

D

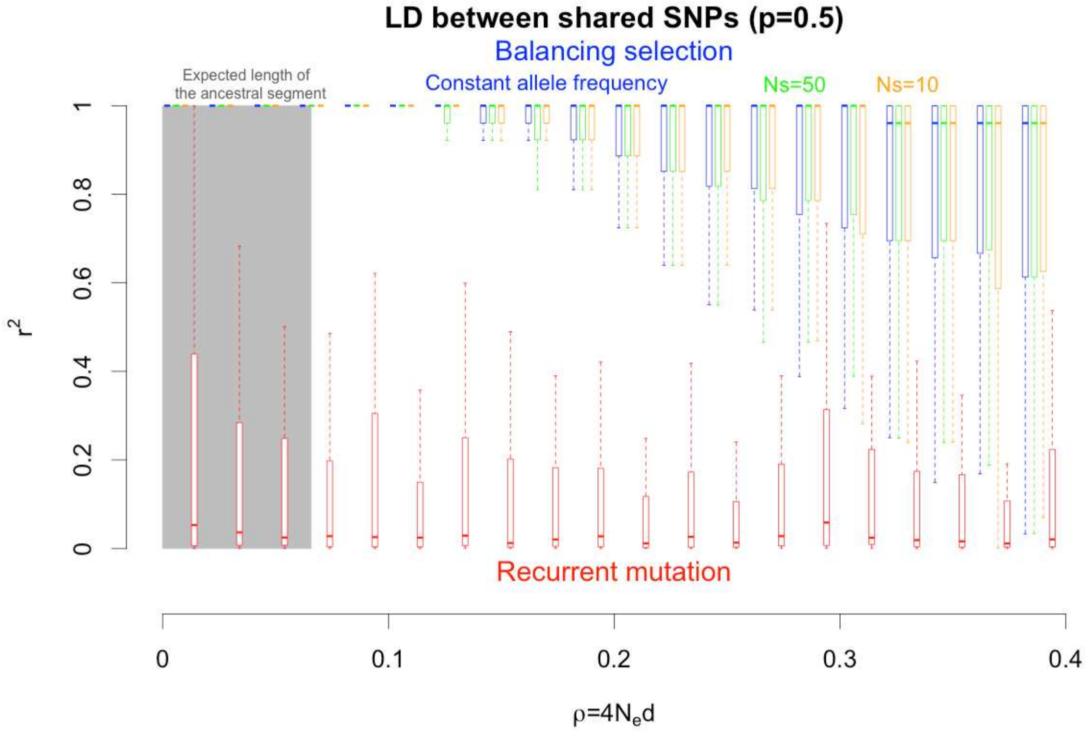
LD between shared SNPs (p=0.5)
Balancing selection
Expected length of the ancestral segment
Constant allele frequency
Ns=50
Ns=10
Recurrent mutation
$r^2$
$\rho=4N_ed$

**Figure S4**: The impact of fluctuations in the selected allele frequencies on the three summary statistics considered. For each statistic, three simulation results (constant allele frequency, *Ns*=10 and *Ns*=50) are shown together with the theoretical predication. (A) Five examples of allele frequency trajectories generated by simulation. Black lines show the actual allele frequencies in each generation. Colored lines are the mean frequencies averaged every 1,000 generations. (B) The distribution of the length of the ancestral segment. (C) The expected coalescent time of two lineages carrying different selected alleles. (D) LD between the SNP under balancing selection and neutral shared SNPs compared with LD between shared SNPs due to recurrent mutations.

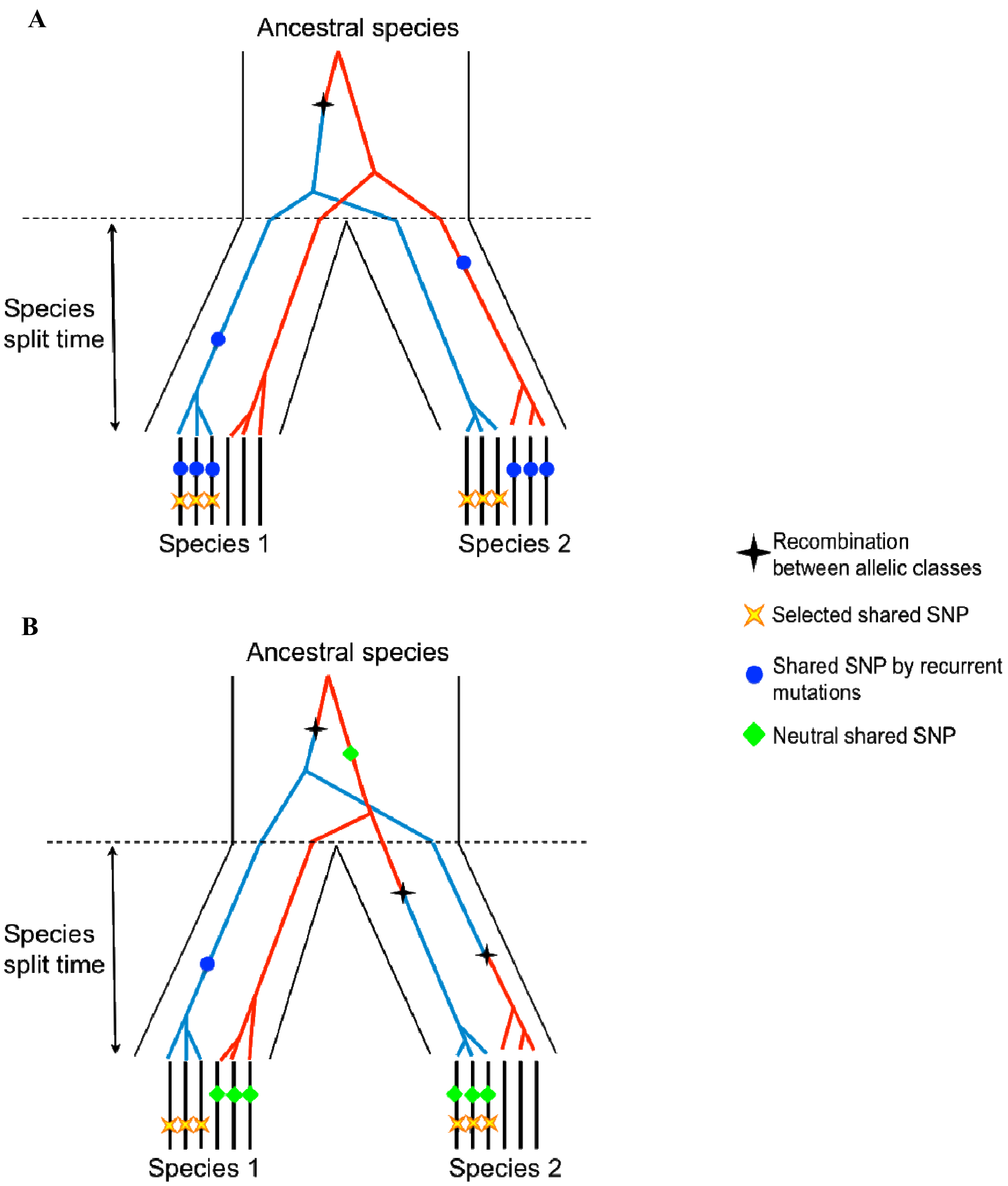


**Figure S5**: Two scenarios that can generate shared polymorphisms in LD but with the opposite phases in the two species. (A) Recurrent mutations on the genealogy of a trans-species polymorphism: independent occurrences of same mutation occur in both species but arise on lineages carrying different selected alleles in the two species. (B) Complex recombination events: two recombination events occur in species 2 before the split, which switch the alleles carried by the two lineages at the selected site in that species.

**Table S1: Summaries of the length of the contiguous ancestral segment**

| | | Length (in bps) | | | |
|---|---|---|---|---|---|
| Split time ($T$) | | 1st Qu. | Median | 3rd Qu. | Mean |
| 100,000 | Approximation | 70 | 171 | 352 | 258 |
| | Simulation | 76 | 183 | 361 | 274 |
| 250,000 | Approximation | 38 | 90 | 182 | 131 |
| | Simulation | 39 | 93 | 188 | 137 |
| 500,000 | Approximation | 21 | 51 | 102 | 73.1 |
| | Simulation | 23 | 55 | 107 | 76.9 |

| | | Length (in bps) | | | |
|---|---|---|---|---|---|
| Recombination rate ($r$) | | 1st Qu. | Median | 3rd Qu. | Mean |
| 1.2cM/Mb | Approximation | 38 | 90 | 182 | 131 |
| | Simulation | 39 | 93 | 188 | 137 |
| 0. 6cM/Mb | Approximation | 75 | 180 | 363 | 262 |
| | Simulation | 72 | 179 | 359 | 266 |
| 0. 3cM/Mb | Approximation | 149 | 359 | 725 | 525 |
| | Simulation | 143 | 361 | 763 | 545 |

| | | Length (in bps) | | | |
|---|---|---|---|---|---|
| Population size ($N_e$) | | 1st Qu. | Median | 3rd Qu. | Mean |
| Not involved | Approximation | 38 | 90 | 182 | 131 |
| 10000 | Simulation | 39 | 93 | 188 | 137 |
| 15000 | Simulation | 39 | 94 | 190 | 140 |
| 20000 | Simulation | 38 | 90 | 181 | 136 |

| | | Length (in bps) | | | |
|---|---|---|---|---|---|
| Allele frequency ($p$) | | 1st Qu. | Median | 3rd Qu. | Mean |
| 0.1 | Approximation | 43 | 101 | 204 | 146 |
| | Simulation | 36 | 87 | 177 | 129 |
| 0.2 | Approximation | 42 | 97 | 197 | 142 |
| | Simulation | 37 | 88 | 179 | 130 |
| 0.3 | Approximation | 40 | 94 | 189 | 140 |
| | Simulation | 37 | 89 | 181 | 131 |
| 0.4 | Approximation | 37 | 96 | 194 | 140 |
| | Simulation | 38 | 90 | 181 | 131 |
| 0.5 | Approximation | 39 | 93 | 188 | 137 |
| | Simulation | 38 | 90 | 182 | 131 |

Parameters used were: $N_e$ =10,000, $N_a$=50,000, $T$=250,000, $r$=1.2cM/Mb and $p$=0.5.

**Table S2: Influence of sample size on the number of neutral shared SNPs and LD among them.**

| Total sample size (number of chromosomes sampled from each class in each species) | Average number of shared neutral SNPs per replicate (100,000 replicates) | | | |
|---|---|---|---|---|
| | All | SNPs in perfect LD with the selected one in species 1 | SNPs in perfect LD with the selected one in species 2 | SNPs in perfect LD with the selected one in both species |
| 4 (1) | 19.022 | 19.022 (100%) | 19.022 (100%) | 19.022 (100%) |
| 20 (5) | 19.690 | 18.884 (95.9%) | 18.865 (95.8%) | 18.154 (92.2%) |
| 40 (10) | 19.767 | 18.657 (94.4%) | 18.666 (94.4%) | 17.726 (89.7%) |
| 100 (25) | 19.798 | 18.333 (92.6%) | 18.353 (92.6%) | 17.162 (86.7%) |
| 200 (50) | 19.811 | 18.140 (91.6%) | 18.128 (91.5%) | 16.811 (84.9%) |

The same number of chromosomes was sampled from each allelic class in each species. Parameters were chosen to be plausible for humans and chimpanzees: $p$=0.5, $T$=20$N_e$, $N_a$= $N_e$, and $T_{BS}$=400 $N_e$. See Supplementary text for further details.

**Table S3: Proportion of ancestral balanced polymorphisms that persist in both species until present**

| | $s$ =0.001 | $s$ =0.005 |
|---|---|---|
| $p$ =0.1 | 0% | 0% |
| $p$ =0.3 | 0% | 74.3% |
| $p$ =0.5 | 29.5% | 85.5% |

We consider a simple demographic model, with parameters plausible for human populations and Western chimpanzees: $T$=250,000, $N_e$=10,000 and $N_a$=50,000. The balanced polymorphism arose 11 $N_a$ generations prior to the split from an initial frequency of $p_0$=0.01. 2000 replicates were generated for each combination of selection strength ($s$) and equilibrium allele frequency ($p$).

**Table S4: Parameter values used in simulations of neutral recurrent mutations.**

**Parameters for both demographic models**

| **Parameter** | **Value** |
|---|---|
| Mutation rate | $1.8\times10^{-8}$ per generation per base pair |
| Recombination rate | 1.2 cM/Mb per generation<br>(sex-averaged mean recombination rate in human genome) |
| Sample size | 50 chromosomes from each species |
| Segment length | 100 kb |
| Proportion of CpG sites | 2% |

**Demographic model with constant population sizes**

| **Parameter** | **Value** |
|---|---|
| Effective population size for humans ($N_h$) | 15,800[a] |
| Effective population size for Western chimpanzees ($N_c$) | 11,100[a] |
| Split time ($T$) | 240,000 generations[b] |
| Effective population size for ancestral species ($N_a$) | 50,000 |

[a] Derived from a mutation rate of $1.8\times10^{-8}$ and the observed heterozygosity
[b] Derived from a split time of 6 Myr, assuming a generation time of 25 years

**Demographic model with bottlenecks**

| **Parameter** | **Value** |
|---|---|
| Effective population size for humans ($N_h$) | 13,900[c] |
| Effective population size for Western chimpanzees ($N_c$) | 12,500[d] |
| Period of the bottleneck for humans | 28-56 kya[c] |
| Period of the bottleneck for Western chimpanzees | 15-35 kya[d] |
| Effective population size for humans during the bottleneck ($N_h'$) | 2,200 |
| Effective population size for Western chimpanzees during the bottleneck ($N_c'$) | 3,500 |
| Split time ($T$) | 240,000 generations |
| Effective population size for ancestral species ($N_a$) | 50,000 |

[c] Based on (Li and Durbin 2011)
[d] Based on (Prado-Martinez et al. 2013)

**Table S5: Percentage of trans-species balanced polymorphisms accompanied by shared neutral SNP(s) in coalescent simulations**

| | Age of the balanced polymorphism | | |
|---|---|---|---|
| **In 1000 replicates** | $20N_e$ | $30N_e$ | $40N_e$ |
| Cases with shared neutral SNP(s) | 65.4% | 89.2% | 93.6% |
| Cases with shared neutral SNP(s) in strong LD with the selected one | 65.3% | 88.6% | 93.4% |
| Average number of shared neutral SNPs in each replicate | 2.47 | 4.10 | 5.64 |

We consider a simple demographic model for human populations and Western chimpanzees ($T$=16$N_e$, $N_a$=5$N_e$) and assume a constant allele frequency of $p$=0.5 and reasonable population mutation rate ($\theta$=1/kb) and population recombination rate ($\rho$=0.5/kb). Each replicate consists of 50 chromosomes from each species.
